# Plating versus elastic stable intramedullary nailing for displaced pediatric midshaft clavicular fractures

**DOI:** 10.1186/s10195-022-00659-2

**Published:** 2022-08-22

**Authors:** Pan Hong, Ruikang Liu, Saroj Rai, Renhao Ze, Xin Tang, Jin Li

**Affiliations:** 1 Department of Orthopaedic Surgery, Union Hospital, Tongji Medical College, Huazhong University of Science and Technology, Wuhan, 430022 China; 2 Department of Endocrinology, Union Hospital, Tongji Medical College, Huazhong University of Science and Technology, Wuhan, China; 3 Department of Orthopaedics and Trauma Surgery, Blue Cross Hospital, Tripureswor, 44600 Kathmandu Nepal; 4 Department of Orthopaedics and Trauma Surgery, Karama Medical Center, Dubai Investment Park Br, Dubai, UAE

**Keywords:** Clavicle fracture, Adolescent, Elastic stable intramedullary nail

## Abstract

**Introduction:**

Traditionally, operative treatment for displaced midshaft clavicle fractures in adolescents has been achieved by using a plate and screws. However, a minimally invasive trend has led surgeons to use the elastic stable intramedullary nail (ESIN) for displaced midshaft clavicle fractures. This study aims to compare the clinical outcomes of adolescent patients who were operated on with an ESIN vs. a plate.

**Methods:**

All patients aged between 10 and 14 years with displaced midshaft clavicle fractures who were operated on at our institute between January 2014 and January 2018 were reviewed retrospectively. The preoperative data, including baseline information on the patients, and types of surgical procedure were collected from the hospital database. The postoperative data, including clinical outcome and complications, were collected during the follow-up visits. Clinical outcome was evaluated during outpatient visits using the American Shoulder and Elbow Surgeons (ASES) score. The scar problem was evaluated according to the Scar Cosmesis Assessment and Rating (SCAR) scale.

**Results:**

A total of 73 patients were included. Patients were categorized into two groups: ESIN (*n* = 45; 27 males, 18 females) and plate (*n* = 28; 17 males, 11 females), according to surgical technique. The average age of the patients in the ESIN group was 12.2 ± 1.5 years, and that in the plate group was 12.2 ± 1.4 years. The ESIN group presented significantly less operative time (31.1 vs. 59.8 min), a shorter hospital stay (1.5 vs. 2.5 days), and a smaller incision (2.4 vs. 5.4 cm) as compared to the plate group (*P* < .001). The rate of scar concern was much higher in the plate group (71.4%) than the ESIN group (22.2%) (*P* < .001). There was no statistically significant difference in shoulder function between the ESIN group and the plate group at different time points.

**Conclusion:**

A conservative approach remains the first choice for a pediatric clavicle fracture. Both the ESIN and the plate are safe and effective treatment methods for displaced midshaft clavicle fractures in adolescents. The ESIN is superior to the plate given its shorter operative time, shorter hospital stay, lower rate of scar concern, and easier implant removal.

**Level of evidence:**

III, retrospective observational study.

## Introduction

Clavicle fracture is one of the most common fractures in the pediatric population and accounts for about 10–15% of all fractures [1]. The midshaft is the most commonly involved anatomical location [2]. In adults, there has been an increasing trend for operative interventions, as they have been reported to give better functional outcomes [3–5]. However, the management of displaced midshaft clavicle fractures in adolescents remains poorly investigated [6–8].

There is a debate about surgical intervention in clavicle fractures in adolescents, as these fractures heal fast and have great remodeling potential. The absolute indications for surgery in adolescents are an open clavicle fracture, a floating shoulder, and associated neurovascular injuries [9]. Still, surgical treatment is gaining popularity among surgeons for adolescents who require early functional recovery and have a high activity level [9, 10].

Traditionally, operative treatment has been achieved by using a plate and screws [7, 10, 11]. Recently, intramedullary fixation has gained increased interest [12, 13]. To the author's knowledge, there is no head-to-head comparison between the elastic stable intramedullary nail (ESIN) and plate fixation for displaced midshaft clavicle fractures in adolescents. This study aims to compare the clinical outcomes of adolescent patients operated on with the ESIN vs. a plate for displaced midshaft clavicle fractures. We hypothesized that the ESIN would deliver better clinical outcomes than plate fixation.

## Materials and methods

This study was approved by the Ethics Committee of Tongji Medical College, Huazhong University of Science and Technology. Written consent was obtained from the patient's legal guardians.

### Inclusion and exclusion criteria

Inclusion criteria were (1) patients aged between 10 and 14 years with (2) a fully displaced midshaft clavicle fracture with (3) no or minimal comminution and (4) bone shortening of over 1.0 cm.

Exclusion criteria were (1) a pathological fracture, (2) metabolic disease, (3) a neuromuscular disorder, (4) an open fracture, (5) overweight patients (50 kg or more), (6) a previous history of ipsilateral clavicle fracture, and (7) a follow-up of less than 24 months.

We further divided the patients into subgroups A and B by fracture type using the AO/OTA classification: simple fracture (type A) and wedge fracture (type B) [14]. Multi-fragmentary fractures (type C) were excluded from our study.

### Data extraction

The preoperative data, including sex, age, body weight, time from injury to surgery, and types of surgical procedure, were collected from the hospital database. The postoperative data, including clinical outcomes and complications, were collected during the follow-up visits.

The visual analogue scale (VAS) was used to evaluate the postoperative pain, and the American Shoulder and Elbow Surgeons (ASES) score was used to evaluate clinical outcomes [15]. Scar concern was evaluated according to the Scar Cosmesis Assessment and Rating (SCAR) scale [16, 17].

### Treatment procedure

**Fig. 1:**
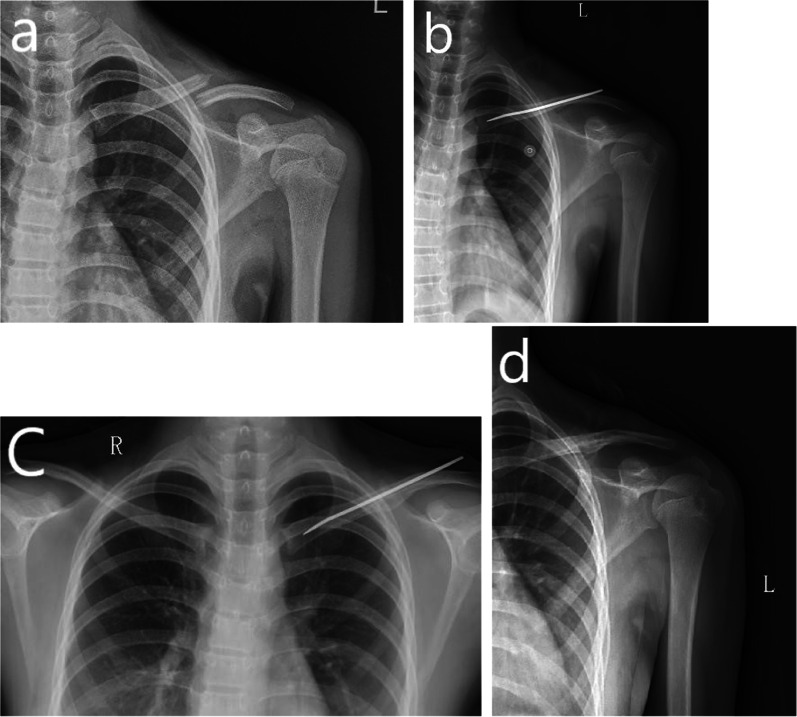
Ten-year-old boy with a right clavicle fracture treated with an ESIN. **a** AP view of the right clavicle before surgery. **b** AP view of the right clavicle after surgery. **c** AP view of the right clavicle at the 1-month follow-up visit after surgery. **d** AP view of the right clavicle after hardware removal. **e** AP view of the right clavicle at the 2-month follow-up visit after hardware removal. *AP* Anterior posterior

**Fig. 2:**
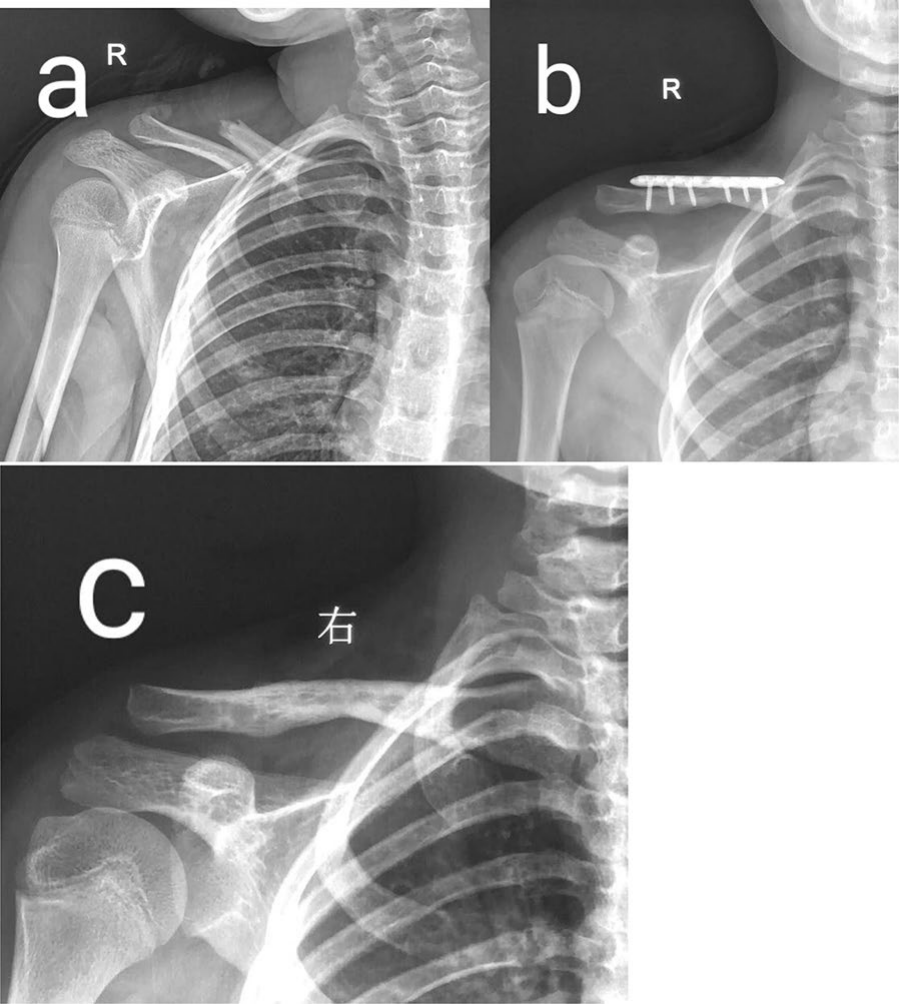
Eleven-year-old boy with a right clavicle fracture treated with a plate. **a** AP view of the right clavicle before surgery. **b** AP view of the right clavicle after surgery. **c** AP view of the right clavicle after hardware removal

Patients were divided into an ESIN group and a plate group. Regarding the surgical technique used in the ESIN group (see Fig. 1), closed reduction usually requires excessive irradiation, so a mini-incision (1.5–3.0 cm) was usually performed to expose the fracture site. A single nail of appropriate diameter (2.5–3.5 mm) was chosen, and reaming was necessary with smooth K-wire of the same diameter. Regarding the surgical technique of the plating group (see Fig. 2), an incision of length 4 to 7 cm was made along the clavicle, and a plate with six to eight holes was used to fixate the fracture with at least four to six cortices on either side of the fracture line. However, as this was a retrospective cohort study, the allocation process depended upon the surgeon’s experience and preference. Therefore, the two groups had different numbers of participants.

An arm sling was used as postoperative care to immobilize the shoulder for 1 to 2 weeks. Active shoulder exercises were encouraged as soon as the pain is reduced. Patients were followed up every 2–3 months at the outpatient clinic. Sports activities were allowed according to the radiographic and clinical manifestation. Hardware removal was performed routinely in all patients. ESIN removal was usually scheduled for about 6–9 months after surgery, and removal of the plate was scheduled for about 9–12 months after the primary surgery.

### Statistical analysis

The SPSS statistical package (SPSS version 19.0; SPSS Inc., Chicago, Illinois, USA) was used for statistical analysis. The categorical data were analyzed using the Chi-square (*χ*^2^) test, and the continuous data were analyzed using Student’s *t*-test. Fisher’s exact test was used when their were fewer subjects in the groups of interest. Data are presented as mean (± SD), median (± interquartile range), or *n* (%), as appropriate. *P* < 0.05 was considered to indicate a significant different.

## Results

A total of 84 patients aged between 10 and 14 years with displaced midshaft clavicle fractures who were operated on in our hospital between January 2014 and January 2018 were reviewed retrospectively. Eleven of them were excluded because they had a pathological fracture (*n* = 5) or an open fracture (*n* = 3), were overweight (*n* = 2), or had incomplete medical records (*n* = 1). Patients were categorized into the ESIN and plate groups. A total of 45 patients, including 27 males and 18 females, were included in the ESIN group, and 28 patients, including 17 males and 11 females, were included in the plate group. As shown in Table 1, there was no significant difference between the ESIN group and the plate group in terms of sex, age, operated side, body weight, and time from injury to surgery.

**Table 1:** Patient demographic

Parameters	ESIN (*N *= 45)	Plate (*N *= 28)	*P* value
Sex			
Male	27	17	0.96
Female	18	11	
Side			
Left	18	11	0.96
Right	27	17	
Age (years)	12.2 ± 1.5 (11, 13)	12.2 ± 1.4 (11, 13)	0.97
Body weight (kg)	38.9 ± 5.2 (34.6, 43.1)	39.1 ± 5.3 (34.7, 42.9)	0.87
Time from injury to surgery (days)	2.0 ± 0.9 (1, 3)	2.2 ± 0.8 (1, 3)	0.43
Follow-up (months)	28.7 ± 4.2 (25.0, 32.3)	29.6 ± 4.6 (25.7, 32.9)	0.74

Values of continuous variables are described as mean ± SD (interquartile range)

*ESIN* elastic stable intramedullary nail

Perioperative outcomes of surgery are shown in Table 2, and all the patients were followed up for 24 months or more. The ESIN group demonstrated a significantly shorter operative time (31.1 vs. 59.8 min), a shorter hospital stay (1.5 vs. 2.5 days), and a shorter incision length (2.4 vs. 5.4 cm) than the plate group (*P* < 0.001). Significant alleviation of pain was noticed in both groups after the surgery, but the pain response showed no significant difference between the two groups at different time points after surgery.

**Table 2:** Perioperative outcomes of surgery

	ESIN (*N *= 45)	Plate (*N* = 28)	*P* value
Operative time (min)	31.1 ± 0.5 (30, 31)	59.8 ± 6.6 (54, 65)	< 0.001^*^
Length of stay (days)	1.5 ± 0.8 (1, 2)	2.5 ± 0.8 (2, 3)	< 0.001^*^
Length of incision (cm)	2.4 ± 0.5 (2.0, 2.7)	5.4 ± 0.9 (4.7, 6.1)	< 0.001^*^
VAS			
Before surgery	7.0 ± 0.8 (7, 8)	7.0 ± 0.8 (7, 8)	0.86
1st day	4.8 ± 0.8 (4, 5)	5.0 ± 0.8 (5, 6)	0.39
2nd day	2.8 ± 0.8 (2, 3)	3.0 ± 0.8 (2, 4)	0.38
3rd day	2.5 ± 0.7 (2, 3)	2.8 ± 0.6 (3, 3)	0.18

Values of continuous variables are described as mean ± SD (interquartile range)

*VAS* visual analogue scale, *ESIN* elastic stable intramedullary nail

^*^P ≤ 0.05

Postoperative and follow-up outcomes are shown in Table 3. Two patients (7.1%) in the plate group suffered a refracture after implant removal. The rate of implant prominence was higher in the ESIN group (44.4%) than in the plate group (32.1%). The rate of surgical site infection (SSI) was low in the ESIN group (4.4%) and the plate group (7.1%). Moreover, the SCAR scale was higher in the plate than in the ESIN group at all time points (*P* < 0.001), and the rate at which cosmetic counsel was sought due to esthetic concerns was also much higher in the plate group (71.4%) than in the ESIN group (22.2%) (*P* < 0.001). The ASES score showed no significant difference between the two groups at any time point.

**Table 3:** Postoperative and follow-up outcomes

	ESIN (*N* = 45)	Plate (*N* = 28)	*P* value
Complication			
Loss of reduction	0	0	1
Nonunion	0	0	1
Refracture	0	2 (7.1%)	0.08
Implant prominence	20 (44.4%)	9 (32.1%)	0.30
SSI	2 (4.4%)	2 (7.1%)	0.60
SCAR scale			
3rd month	3.5 ± 1.5 (2, 4)	7.7 ± 2.3 (6, 9)	< 0.001^*^
Before hardware removal	3.2 ± 1.8 (2, 4)	8.2 ± 2.3 (7, 10)	< 0.001^*^
3rd month after removal	2.9 ± 1.6 (2, 4)	8.3 ± 2.2 (7, 10)	< 0.001^*^
Cosmetic counsel^#^	10 (22.2%)	20 (71.4%)	< 0.001^*^
ASES score			
3rd month	90.4 ± 2.7 (88, 92)	90.8 ± 2.5 (89, 92)	0.47
6th month	92.8 ± 2.2 (91, 94)	93.1 ± 2.4 (91, 95)	0.56
Last follow-up	94.5 ± 2.8 (92, 96)	95.1 ± 2.7 (93, 96)	0.36

Values of ontinuous variables are described as mean ± SD (interquartile range)

*ESIN* elastic stable intramedullary nail, *SSI* surgical site infection, *SCAR* Scar Cosmesis Assessment and Rating, *ASES* American Shoulder and Elbow Surgeons

^*^P ≤ 0.05

^#^Cosmetic counsel was sought due to esthetic concerns

## Discussion

Both the ESIN group and the plate group produced satisfactory clinical outcomes for displaced midshaft clavicle fractures in adolescents. The ESIN is superior to the plate, given that it permits a shorter operative time, a shorter hospital stay, less esthetic concern, and easier implant removal.

Operative management is gaining popularity for clavicle fractures in adults because of better clinical outcomes [5]. In children, nonoperative management usually results in good functional outcomes [18, 19]. However, surgical treatment for teenagers seems to have been popular over the past 10 years [20]. Especially for adolescents that demand early functional recovery and have a high activity level, surgery is an alternative choice [7, 9, 10, 21]. However, nonoperative management should remain the gold standard when treating pediatric and adolescent clavicle fractures [22, 23]. Therefore, operative management should be carried out discretely. Besides, the pros and cons must be explained thoroughly to the parents and the patients before undertaking the surgery. Evidently, the ESIN demonstrated the advantages of a minimally invasive approach as it allowed a smaller incision, faster surgery, and a shorter hospital stay than the plate. Besides, the removal of the ESIN was easier than the removal of the plate. In contrast, the ESIN has a high risk of implant prominence, and patients are immobilized in an arm sling for 1–2 weeks after the operation to ensure recovery and stability. Besides, plating leads to better anatomical reduction and stronger fixation.

In our study, the clinical outcomes in both groups were satisfactory, consistent with previous reports [24–28]. There were no statistically significant differences in terms of shoulder function and serious complications between the two groups. Also, there was no case of nonunion or malunion in both groups.

Previous studies reported early complications such as nail breakage, bending, threatened skin perforation, and clavicle shortening in the ESIN group [24, 27, 29]. However, these complications were not significant in our study, possibly because of the routine immobilization in an arm sling for 1 to 2 weeks, good patient compliance, and the exclusion of multi-fragmentary fractures from our study. Besides, the refracture rates of both groups were low, consistent with previous reports, and two patients suffered from a refracture resulting from an accidental fall after plate removal [11, 19, 28, 29].

As shown in “Results” section, the SCAR scale was much higher in the plate group than in the ESIN group at different time points. Besides, the percentage of the patients who sought cosmetic counsel due to esthetic concerns was higher in the plate group (71.4%) than in the ESIN group (22.2%).

We undertook a retrospective investigation, so our findings should be interpreted with caution. Firstly, the process of allocating patients to either the ESIN group or the plate group depended partly on the preference of the surgeon in charge, and this strategy may have caused allocation bias. Besides, preoperative radiographic parameters, including displacement and angulation, were not recorded and analyzed. In the follow-up visits, certain morphological abnormalities of the clavicle were noticed in the radiograph but not recorded and analyzed, because the gross appearance seemed normal. Moreover, patients treated with nonoperative methods were not included because the purpose of this study was to discuss the pros and cons of operative choices. Furthermore, there was an insignificant difference in complications between the two groups due to the small number of included patients.

## Conclusion

A conservative approach remains the first choice for pediatric clavicle fracture. Both the ESIN and a plate are safe and effective treatment methods for displaced midshaft clavicle fractures in adolescents. The ESIN is superior to the plate, given that it permits a shorter operative time, a shorter hospital stay, less scar concern, and easier implant removal.

## Abbreviations

ESIN: Elastic stable intramedullary nail
ASES: American Shoulder and Elbow Surgeons
SCAR: Scar Cosmesis Assessment and Rating

## Declarations

### Availability of data and materials

Data sharing is not applicable to this article as no datasets were generated or analyzed during the current study.
